# Immunohistochemistry of sarcolemmal membrane-associated proteins in formalin-fixed and paraffin-embedded skeletal muscle tissue: a promising tool for the diagnostic evaluation of common muscular dystrophies

**DOI:** 10.1186/s13000-017-0610-y

**Published:** 2017-02-20

**Authors:** Chinnawut Suriyonplengsaeng, Charungthai Dejthevaporn, Chaiyos Khongkhatithum, Suda Sanpapant, Nattha Tubthong, Koset Pinpradap, Nippa Srinark, Jariya Waisayarat

**Affiliations:** 1 Department of Pathology, Faculty of Medicine Ramathibodi Hospital, Mahidol University, Bangkok, 10400 Thailand; 2 Department of Anatomy, Faculty of Science, Mahidol University, Bangkok, 10400 Thailand; 3 Division of Neurology, Department of Medicine, Faculty of Medicine, Ramathibodi Hospital, Mahidol University, Bangkok, 10400 Thailand; 4 Department of Pediatrics, Faculty of Medicine Ramathibodi Hospital, Mahidol University, Bangkok, 10400 Thailand

**Keywords:** Muscular dystrophy, Formalin-fixed and paraffin-embedded, Snap frozen section, Immunohistochemistry, Muscle biopsy, Dystrophin, Sarcoglycan, Dystroglycan, Dysferlin, Dystrophinopathy

## Abstract

**Background:**

The analysis of fresh frozen muscle specimens is standard following routine muscle biopsy, but this service is not widely available in countries with limited medical facilities, such as Thailand. Nevertheless, immunohistochemistry (IHC) analysis is essential for the diagnosis of patients with a strong clinical suspicion of muscular dystrophy, in the absence of mutations detected by molecular genetics. As the successful labelling of sarcolemmal membrane-associated proteins in formalin-fixed and paraffin-embedded (FFPE) muscle sections using IHC staining has rarely been described, this study aimed to develop a reproducible IHC method for such an analysis.

**Methods:**

Thirteen cases were studied from the files of the Department of Pathology, Mahidol University. Diagnoses included three Duchenne muscular dystrophy (DMD), one Becker muscular dystrophy (BMD), one dysferlinopathy, and several not-specified muscular dystrophies. IHC was performed on FFPE sections at different thicknesses (3 μm, 5 μm, and 8 μm) using the heat-mediated antigen retrieval method with citrate/EDTA buffer, followed by an overnight incubation with primary antibodies at room temperature. Antibodies against spectrin, dystrophin (rod domain, C-terminus, and N-terminus), dysferlin, sarcoglycans (α, β, and γ), and β-dystroglycan were used. Frozen sections were tested in parallel for comparative analysis.

**Results:**

Antibodies labelling spectrin, dystrophin (rod domain and C-terminus), dysferlin, sarcoglycans (α, β, and γ), and β-dystroglycan clearly exhibited sarcolemmal staining in FFPE sections. However, staining of FFPE sections using the antibody directed against the N-terminus of dystrophin was unsuccessful. The absence of labeling for dystrophins and dysferlin in FFPE sections was documented in all three DMD patients and the dysferlinopathy patient. The BMD diagnosis could not be made using IHC in FFPE sections alone because of a lack of staining for the dystrophin N-terminus, indicating a limitation of this method.

**Conclusions:**

We developed a reliable and reproducible IHC technique using FFPE muscle. This could become a valuable tool for the diagnosis of some muscular dystrophies, dystrophinopathies, sarcoglycanopathies (LGMD2D, LGMD2E, and LGMD2C), and dysferlinopathy, especially in situations where the analysis of fresh frozen muscle samples is not routinely available.

## Background

Muscular dystrophies (MDs) are various inherited diseases caused by the absence or dysfunction of proteins essential for myofibre stability, leading to progressive muscular destruction and muscle weakness [1]. MDs encompass Duchenne muscular dystrophy (DMD), Becker muscular dystrophy (BMD), congenital muscular dystrophy, myotonic muscular dystrophy, Emery–Dreifuss muscular dystrophy, facioscapulohumeral dystrophy, oculopharyngeal muscular dystrophy, scapuloperoneal dystrophy, distal myopathies, and limb-girdle muscular dystrophies (LGMDs). LGMDs are further classified into autosomal dominant (LGMD1) and autosomal recessive (LGMD2) forms. MDs exhibit diverse inheritance patterns and manifest with variable phenotypes. Each disorder varies in its age of onset, severity, distribution of affected muscles, pattern of inheritance, and affected muscle groups and other organs. The dystrophinopathies, DMD and BMD, are the most common MDs, and were the first to be documented in the medical literature [2–4].

Considerable advances have been made in recent years towards the identification of genetic mutations as well as the characterization of novel proteins involved in MDs [5–9]. Both DMD and BMD are caused by mutations in the dystrophin gene, located on chromosome Xp21.2, resulting in alterations of dystrophin proteins. A greater understanding of the heterogeneous genetic basis and subsequent alterations of protein expression in each form of LGMD is also being achieved [10]. The absence of dysferlin causes dysferlinopathy, while defects in α-, β-, γ-, or δ-sarcoglycan (SG) genes cause LGMD2D, LGMD2E, LGMD2C, and LGMD2F, respectively. LGMDs 2C–F are collectively grouped as sarcoglycanopathies, with LGMD2D being the most common type.

It is difficult to identify the type of MD based solely on histological and histochemical findings [11], and a definitive diagnosis requires immunohistochemical (IHC) analysis to assess the loss or deficiency of sarcolemmal membrane-associated proteins. This technique is especially important for the evaluation of muscle biopsies from patients with a high clinical suspicion of MD in whom no genetic mutation has been detected. The IHC analysis of fresh frozen muscle specimens remains the standard method for the examination of proteins altered in MD patients using commercial antibodies suitable for use with fresh muscle tissue [12, 13]. The parallel analysis of sarcolemmal integrity, by monitoring proteins such as spectrin or caveolin-3, is also necessary when evaluating sarcolemmal membrane-associated proteins [11–13]. It is important to note that IHC analysis is complementary to histology and histochemistry and should always be interpreted in conjunction with the clinical context and morphological studies. In addition to IHC staining, proteins of fresh muscle tissue samples can be further examined using immunoblot analysis (western blotting) in some clinical circumstances [14].

Although the snap frozen technique has many advantages, the equipment required for the production of fresh frozen sections is not widely available, especially in medical institutions in developing countries. In provincial pathology laboratories with limited budgets, fresh specimen storage at low temperatures, expensive equipment and materials, and the high maintenance costs of freezers are major financial obstacles for performing the analysis of fresh frozen sections. In Thailand, only Ramathibodi and Siriraj Hospitals, two tertiary medical schools of Mahidol University, provide this service to evaluate muscle pathology [15, 16]. Therefore, neuromuscular patients requiring the analysis of muscle biopsies must be referred to one of these two hospitals. The lack of on-site analysis in other hospitals delays diagnosis and limits proper management of disease. Formalin-fixed paraffin-embedded (FFPE) specimen analysis is more readily available in Thailand and other developing countries, but the successful IHC staining of sarcolemmal membrane-associated proteins in FFPE muscle samples has rarely been described [17–19]. However, the potential benefits of using FFPE samples, especially in pathology units with limited resources, prompted us to develop a sensitive and reproducible IHC method for monitoring sarcolemmal membrane-associated proteins in FFPE muscle samples.

## Methods

This study was approved by the Ethical Committee, Faculty of Medicine Ramathibodi Hospital, Mahidol University. A copy of the approved ethic form is available for review from the Editor-in-Chief of this journal.

### Patient data

The 13 MD cases studied were from the files of the Department of Pathology, Mahidol University. All patients had been diagnosed on the basis of clinical information, histologic findings, and the muscle biopsy immunophenotype using the snap frozen method. The cases included three DMD, one BMD, and one dysferlinopathy. Because of the limited genetic investigation at our institution and the patients’ financial status, a diagnosis of not-specified MD was applied to the remaining eight patients. DMD, BMD, dysferlinopathy, and sarcoglycanopthies were, however, excluded in these eight cases. The details of the cases studied are shown in Table 1.

**Table 1:** Summary of the 13 patients included in this study

Case no.	Age (years)	Sex	Diagnosis
1	48	Female	Muscular dystrophy, not specified
2	4	Female	Muscular dystrophy, not specified
3	15	Male	Dysferlinopathy
4	2	Male	Duchenne muscular dystrophy
5	40	Female	Distal myopathy with rimmed vacuole
6	11	Male	Duchenne muscular dystrophy
7	7	Female	Muscular dystrophy, not specified
8	10	Male	Duchenne muscular dystrophy
9	38	Male	Muscular dystrophy, not specified
10	40	Female	Muscular dystrophy, not specified
11	62	Female	Muscular dystrophy, not specified
12	1 month	Male	Muscular dystrophy, not specified
13	39	Male	Becker muscular dystrophy (absence of dystrophin N-terminus)

### Tissue processing

**Fig. 1:**
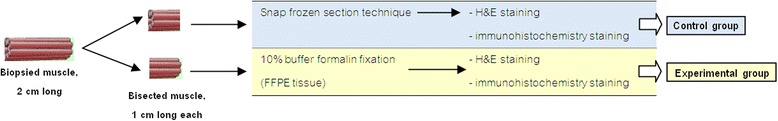
Illustrative flow chart of muscle tissue process used in this study

Each patient underwent open muscle biopsy either from the quadriceps femoris or biceps brachii muscles under local anesthesia. The fresh specimen was immediately sent to the pathology laboratory. A summary of the tissue processing procedure is given in Fig. 1. Each muscle sample was divided into two pieces, with one piece processed using the snap frozen technique, which is the standard method for muscle biopsy analysis. The second piece was fixed in 10% neutral buffered formalin and was further processed into paraffin-embedded blocks.

### Immunohistochemistry

Sections of 3 μm, 5 μm, and 8 μm thickness were cut from each paraffin block. The sections were placed on charged slides (Superfrost Plus slides; Thermo Scientific (Newcastle upon Tyne, United Kingdom)) and dried at 60 °C overnight in a hot air oven. Sections were de-waxed, rehydrated, and underwent a heat-mediated antigen retrieval method using Leica BOND-MAX. They were then incubated in a citrate solution (ER solution 1, Leica) at 100 °C for 40 min, and further incubated with EDTA (ER solution 2, Leica) at 100 °C for 40 min. After dipping the slide in distilled water, endogenous peroxidase blocking was performed by adding 1–2 drops of 5% hydrogen peroxidase, enough to cover the sections, followed by incubation for 30 min. Non-specific background blocking was performed using Bond Primary Antibody Diluent (Leica) with incubation at room temperature for 30 min. Nine primary antibodies, spectrin, dystrophin (rod domain, C-terminus, and N-terminus), dysferlin, SGs (α, β, and γ), and β-dystroglycan, were used in the analysis of muscle samples. Table 2 lists details of the primary antibodies and their concentrations. Antibodies were diluted in Bond Primary Antibody Diluent (Leica) and incubated overnight at 4 °C in a humidified chamber. Immunoreactions were visualised using 3,3'-diaminobenzidine tetrahydrochloride hydrate (DAB) with subsequent counterstaining with Mayer’s haematoxylin using the Bond Polymer Refine Detection System (Leica). Sections were then dehydrated, cleared, and mounted.

**Table 2:** Details of the antibodies used in this study

Antibody	Manufacturer	Clone	Dilution for frozen section	Dilution for FFPE section
Spectrin	Novocastra	RBC2/3D5	1:200	1:20
Dystrophin (rod domain)	Novocastra	Dy4/6D3	1:100	1:20
Dystrophin (C-terminus)	Novocastra	Dy8/6C5	1:100	1:50
Dystrophin (N-terminus)	Novocastra	Dy10/12B2	1:50	1:20
α-sarcoglycan	Novocastra	AD1/20A6	1:200	1:50
β-sarcoglycan	Novocastra	bSarc1/5B1	1:200	1:50
γ-sarcoglycan	Novocastra	35DAG/21B5	1:50	1:20
β-dystroglycan	Novocastra	43DAG/8D5	1:300	1:20
Dysferlin	Novocastra	Ham1/7B6	1:50	1:20

Frozen sections were prepared in parallel as controls. Fresh muscle specimens were rapidly frozen in isopentane (−150 °C) and cooled in liquid nitrogen (−80 °C). Cryostat sections (10 μm) were cut and dried on glass slides at room temperature. No fixation or pretreatment was performed prior to the IHC analysis. Samples were incubated for 40 min with primary antibodies diluted in Bond Primary Antibody Diluent (Leica). Visualisation with DAB and subsequent counterstaining with Mayer’s haematoxylin were performed using the Bond Polymer Refine Detection System (Leica). Sections were then dehydrated, cleared, and mounted.

A non-muscular dystrophy muscle tissue was utilized as a normal control for FFPE and frozen sections. Each case number in the FFPE group was blinded and randomly reordered for IHC interpretation. IHC results using FFPE sections were interpreted in isolation, without clinical information. The final diagnosis was determined by combining the IHC result from the analysis of FFPE muscle sections with the clinical context. A comparison of IHC staining between FFPE and frozen sections was also performed.

## Results

**Fig. 2:**
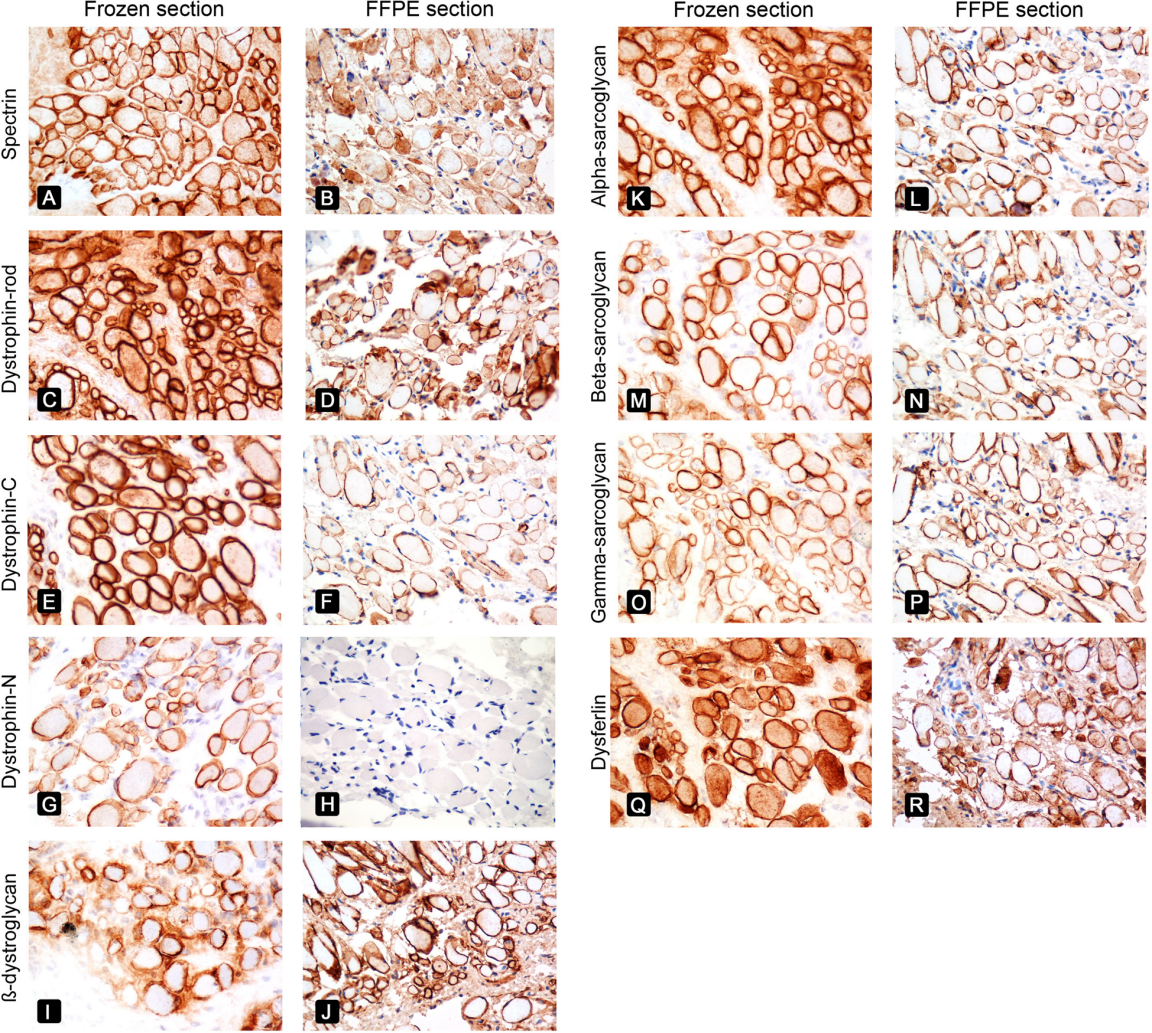
Comparison of IHC results between frozen sections (*left column*) and FFPE sections (*right column*) in patient 12, with not-specified muscular dystrophy. Equivalent sarcolemmal staining for spectrin (**a**, **b**), the rod domain of dystrophin (**c**, **d**), the C-terminus of dystrophin (**e**, **f**), β-dystroglycan (**i**, **j**), α-sarcoglycan (**k**, **l**), β-sarcoglycan (**m**, **n**), γ-sarcoglycan (**o**, **p**), and dysferlin (**q**, **r**) was evident. The N-terminus of dystrophin could not be stained in the FFPE section (**h**) while its expression was apparent in the frozen section (**g**). Original magnification, ×400

IHC results using frozen sections and FFPE sections for each case are summarized in Tables 3 and 4, respectively. The immunopositivity of spectrin in all biopsies indicated well-preserved sarcolemmal integrity, ensuring that false negative results for other sarcolemmal membrane-associated proteins could be ruled out. Clear sarcolemmal staining was observed using antibodies against spectrin, the dystrophin rod domain, the dystrophin C-terminus, SGs (α, β, and γ), β-dystroglycan, and dysferlin in both FFPE and frozen sections (Fig. 2a-r). Although normal membranous staining of the dystrophin N-terminus was achieved in frozen sections (Fig. 2g), it could not be demonstrated in any FFPE sections, even when using a dilution of 1:20 (Fig. 2h, Table 4).

**Table 3:** Immunohistochemical staining analysis of frozen sections for each case

Case	Spectrin	DYS1	DYS2	DYS3	α-SG	β-SG	γ-SG	β-DG	Dysferlin
1	+	+	+	+	+	+	+	+	+
2	+	+	+	+	+	+	+	+	+
3	+	+	+	+	+	+	+	+	−
4	+	−	−	−	+	+	+	+	+
5	+	+	+	+	+	+	+	+	+
6	+	−	−	−	+	+	+	+	+
7	+	+	+	+	+	+	+	+	+
8	+	−	−	−	+	+	+	+	+
9	+	+	+	+	+	+	+	+	+
10	+	+	+	+	+	+	+	+	+
11	+	+	+	+	+	+	+	+	+
12	+	+	+	+	+	+	+	+	+
13	+	+	+	−	+	+	+	+	+

*DYS1* rod domain of dystrophin, *DYS2* C-terminus of dystrophin, *DYS3* N-terminus of dystrophin, *SG* sarcoglycan, *DG* dystroglycan, + positive staining, − negative staining

**Table 4:** Immunohistochemical staining analysis of FFPE sections for each case

Case	Spectrin	DYS1	DYS2	DYS3	α-SG	β-SG	γ-SG	β-DG	Dysferlin
1	+	+	+	Unsuccessful staining	+	+	+	+	+
2	+	+	+		+	+	+	+	+
3	+	+	+		+	+	+	+	−
4	+	−	-		+	+	+	+	+
5	+	+	+		+	+	+	+	+
6	+	−	−		+	+	+	+	+
7	+	+	+		+	+	+	+	+
8	+	−	−		+	+	+	+	+
9	+	+	+		+	+	+	+	+
10	+	+	+		+	+	+	+	+
11	+	+	+		+	+	+	+	+
12	+	+	+		+	+	+	+	+
13	+	+	+		+	+	+	+	+

**Fig. 3:**
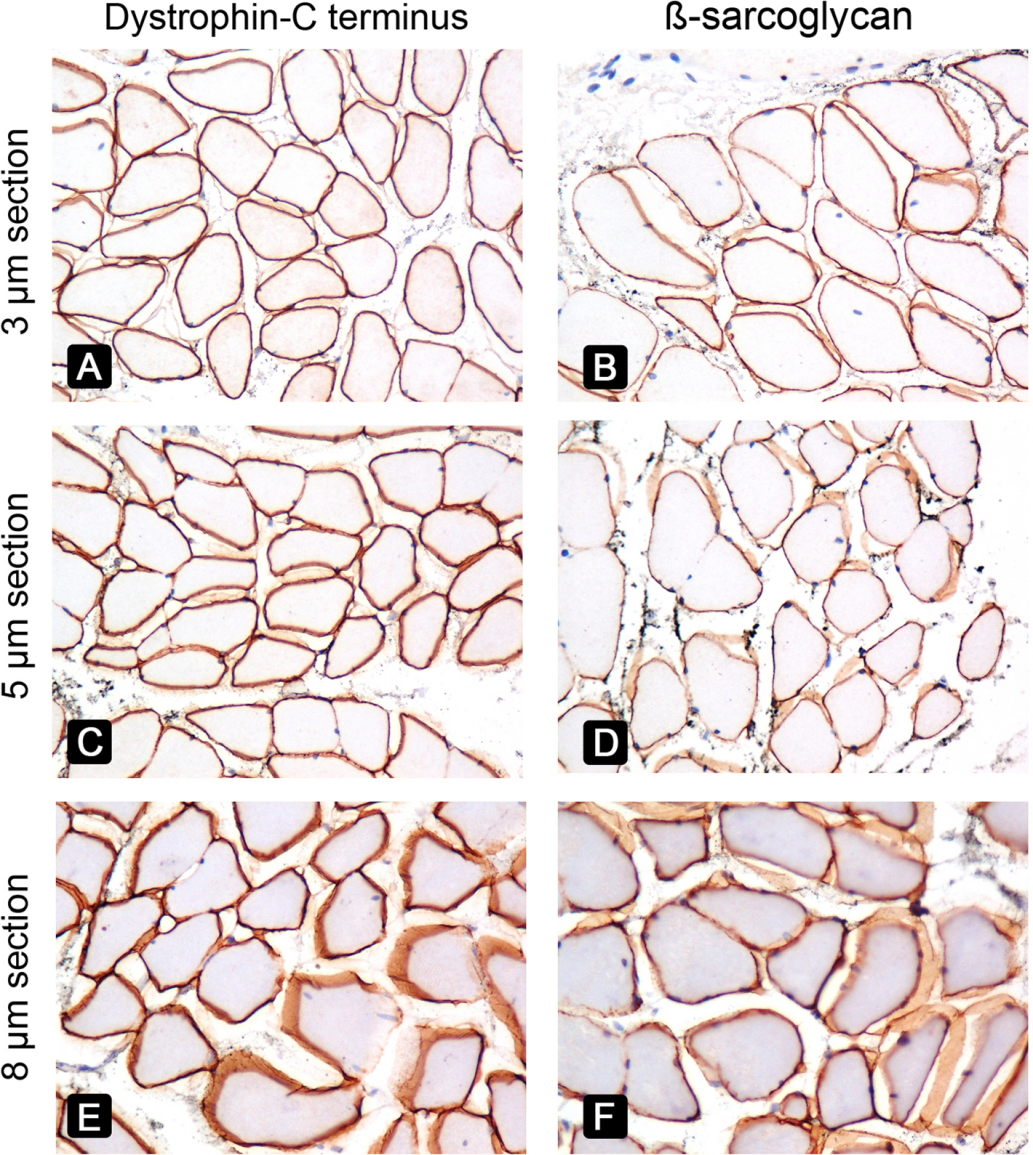
Comparison of varying thicknesses of FFPE sections using normal muscle from a non-MD patient immunostained with antibodies against the C-terminus of dystrophin (*left column*) and β-sarcoglycan (*right column*). FFPE sections were cut into 3-μm (**a**, **b**), 5-μm (**c**, **d**) and 8-μm (**e**, **f**). Original magnification, ×400

**Fig. 4:**
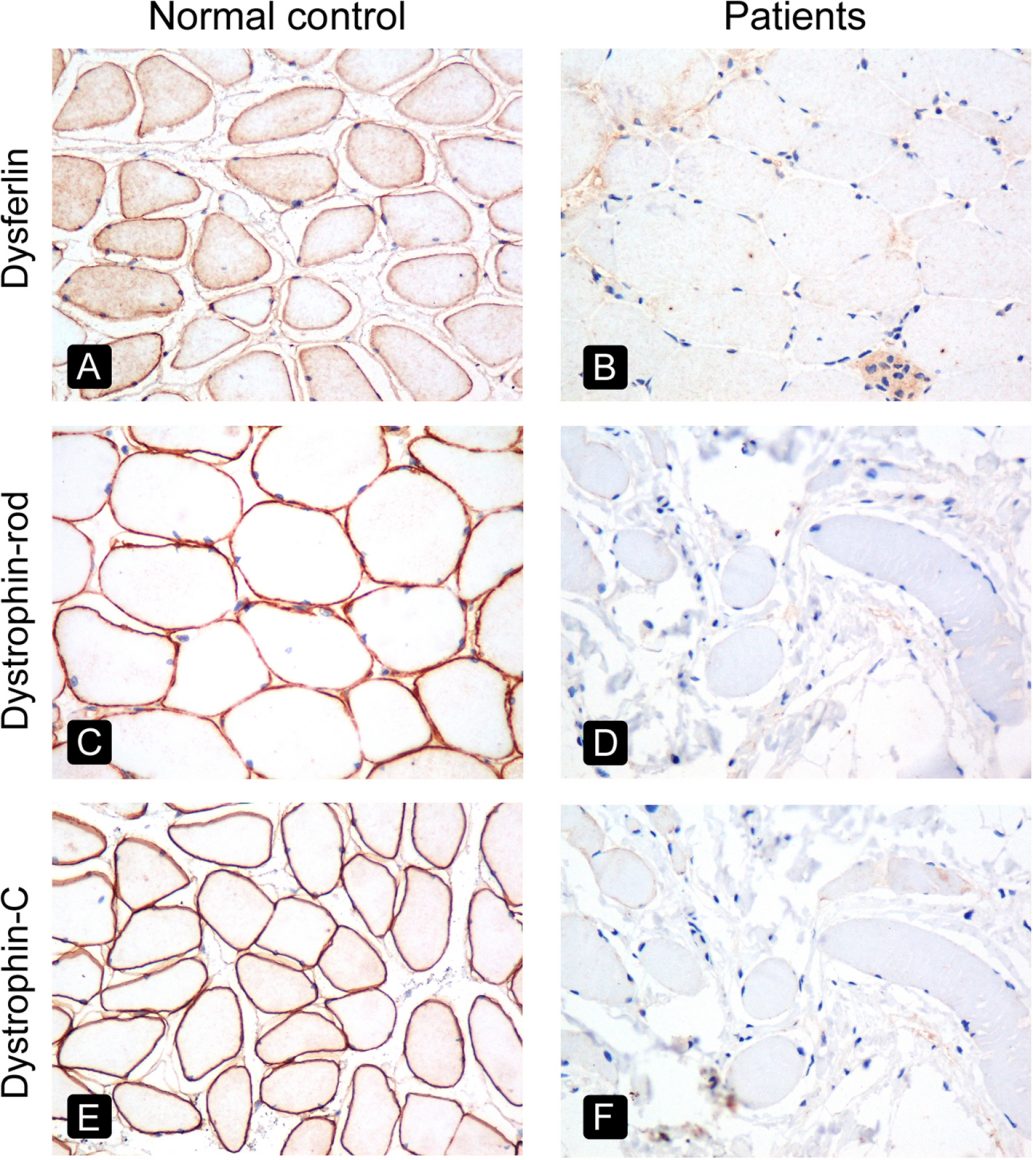
Comparison of FFPE sections of control muscle (*left column*) and muscle biopsy from patients (*right column*). Dysferlin was absent from the FFPE section of patient 3 (**b**), and apparent in control tissue (**a**). The FFPE sections of patient 8 showed no expression of the rod domain (**d**) and C-terminus (**f**) of dystrophin, while control tissue showed positive staining of the rod domain (**c**) and C-terminus (**e**) of dystrophin. Based on the whole clinical picture, histology, and the IHC study of FFPE sections, a diagnosis of dysferlinopathy and DMD were established for patients 3 and 8, respectively. Original magnification, ×400

**Fig. 5:**
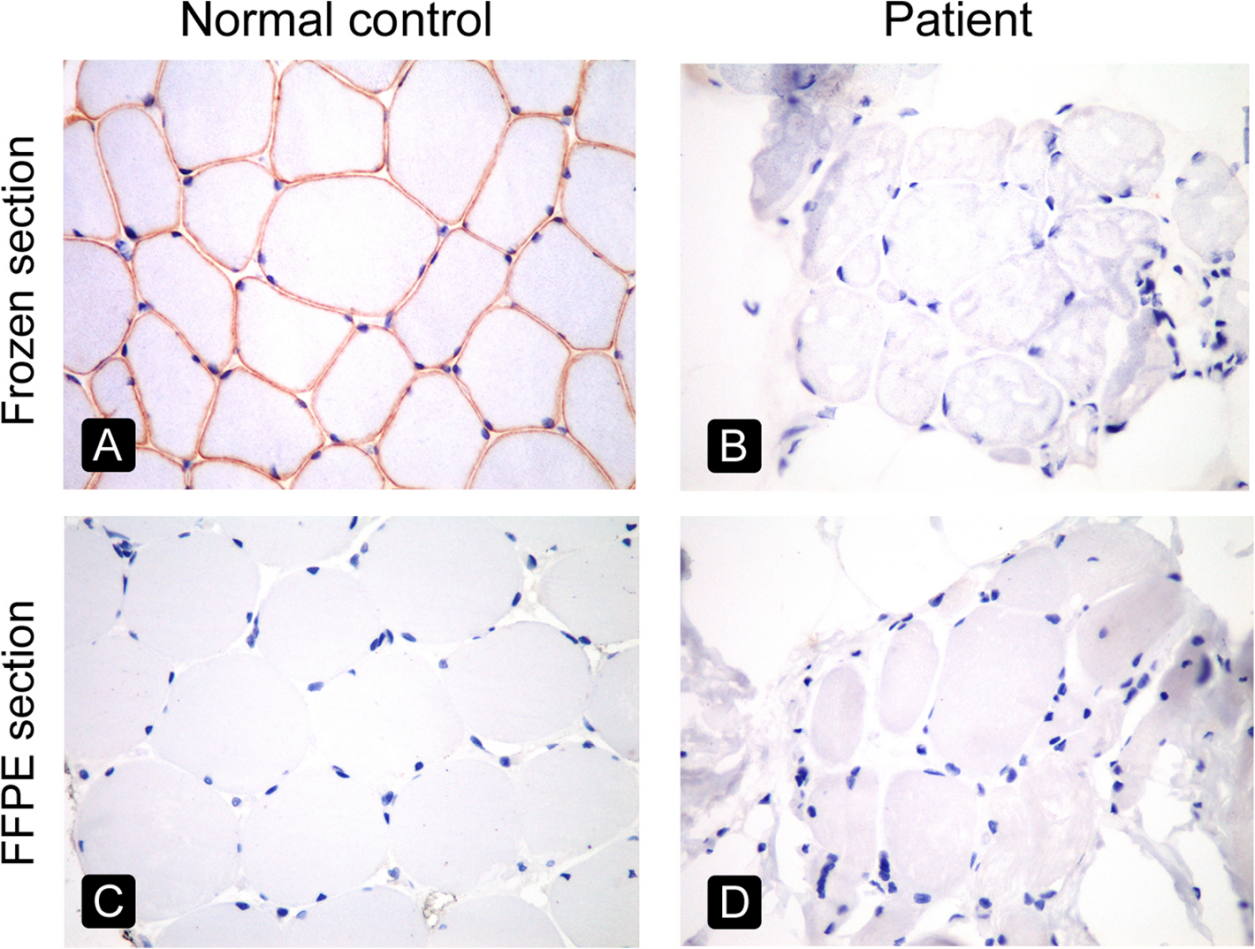
Comparison of frozen sections (*upper row*) and FFPE sections (*lower row*) of control muscle (left column) and muscle biopsy from patient 13 (*right column*). All samples were immunostained with an antibody against the N-terminus of dystrophin. A diagnosis of BMD was established because of the absence of staining for the N-terminus of dystrophin in the frozen section (**b**) and positive staining for the N-terminus of dystrophin in control tissue (**a**), while other sarcolemmal-membrane associated proteins were strongly expressed (data not shown). The FFPE muscle of the patient (**d**) could not be interpreted because of unsuccessful sarcolemmal staining of the control muscle (**c**). Original magnification, ×400

FFPE sections of 3–5 μm gave superior results compared with 8-μm FFPE sections (Fig. 3a-f) because the latter exhibited overlapping sarcolemmal staining from adjacent myofibres (Fig. 3e-f). The absence of labeling for dysferlin and the rod domain and C-terminus of dystrophin in FFPE sections was documented in all three DMD patients (patients 4, 6, and 8) and the dysferlinopathy patient (patient 3) (Fig. 4), allowing a diagnosis to be made using FFPE sections. Patient 13 was diagnosed with BMD based on clinical information, the absence of staining for the dystrophin N-terminus on frozen sections, and strong sarcolemmal staining of the dystrophin rod domain and C-terminus. In contrast, no staining for the dystrophin N-terminus was observed in FFPE muscle from patient 13 or in control muscle from the same glass slide, implying that a true negative result could not be interpreted (Fig. 5). Therefore, the diagnosis of BMD in patient 13 could not be made using IHC of FFPE sections, representing a limitation of this method in the present study.

## Discussion

In this study, we demonstrate a reliable and reproducible IHC technique, with heat-mediated antigen retrieval, for FFPE muscle specimens using antibodies to spectrin, the dystrophin rod domain and C-terminus, SGs (α, β, and γ), β-dystroglycan, and dysferlin. We suggest that this method could become a valuable tool for the diagnosis of common MDs such as DMD, sarcoglycanopathies (LGMD2D, LGMD2E, and LGMD2C), and dysferlinopathy. It will be especially useful in situations where the fresh frozen technique or genetic investigations are not routinely available. However, the unsuccessful staining of the N-terminus of dystrophin in FFPE muscle shows the limitations of this method for the diagnosis of BMD patients. Moreover, while IHC results are an important component of MD diagnosis, we suggest that they should be correlated with clinical information and histopathological findings such as dystrophic changes in muscle biopsy. Additionally, to prevent the misinterpretation of a secondary reduction in IHC staining as a primary defect, a panel of markers for sarcolemmal membrane-associated proteins is required.

The diagnostic evaluation of dystrophinopathy is proposed as follows: if staining for both the rod domain and C-terminus of dystrophin are absent in FFPE muscle sections, a diagnosis of DMD can be made. However, caution should be exercised in the case of positive labelling of the rod domain and C-terminus of dystrophin because although DMD can be confidently ruled out, BMD cannot be definitively excluded.

The successful IHC immunolabeling of α-, β-, and γ-SGs in this study indicates that alterations in the expression of α-, β-, or γ-SGs can be used to clarify the MD type in cases where the clinical diagnosis of LGMD2D, LGMD2E, or LGMD2C is unclear. Indeed, the intense immunolocalisation of one SG protein in FFPE muscle sections can exclude a defect in the gene encoding that specific SG protein. However, mutations in a single sarcoglycan gene not only diminish the expression of the protein encoded by that gene, but may also lead to a secondary reduction of other SGs. Therefore, the absence or strongest reduction of one SG from IHC analysis typically indicates the true gene defect [20], but no immunostaining pattern is considered to be specific for any single sarcoglycan gene mutation [21]. Additionally, mutations of β- or δ-sarcoglycan genes often result in a total absence of all SGs in IHC analysis [20, 22, 23]. Although IHC for SGs appears to be very sensitive for detecting sarcoglycanopathies, it is not exclusively diagnostic because secondary deficiencies of SG expression can be present in other MDs, such as DMD or BMD [20, 21]. The inter-relationships between proteins affected in MDs mean that the analysis of a panel of IHC markers is essential for a definitive diagnosis.

Evaluating the expression of dysferlin in muscle tissue is necessary for the diagnosis of adolescents or young adults who initially present with proximal muscle weakness, clinically suspicious for LGMD2B, or with distal muscle weakness and the inability to walk on tip-toe, raising the possibility of Miyoshi myopathy. These entities are grouped as dysferlinopathies and are caused by mutations in the dysferlin gene on chromosome 2p13. Analysis of muscle biopsy for dysferlinopathy not only demonstrates dystrophic changes, but also shows perimysial and perivascular lymphocytic infiltrates, morphologically mimicking of inflammatory myopathy. The absence of the dysferlin protein can be confirmed by IHC in FFPE muscle, as demonstrated in patient 3 (Fig. 4a–b). Indeed, our study is the first to report IHC of dysferlin in FFPE sections. The observed intensity of IHC staining for dysferlin was consistently positive and distinct, which appears to be an improvement over existing commercially available antibodies for dysferlin that do not always provide a clear IHC result [12].

LGMD2A or calpainopathy is the most common form of LGMD2. A previous demonstration of the complete absence of calpain-3 by IHC using fresh muscle tissue processed with the snap frozen technique was found to be 100% specific for calpainopathy [24]. Unfortunately, calpain-3 expression could not be examined in the present study because calpain-3 antibody is not commercially available in Thailand. Similarly, we did not perform IHC against merosin, fast myosin heavy chain, slow myosin heavy chain, or δ-sarcoglycan because of the lack of commercially available antibodies. Successful IHC for these proteins in FFPE muscle sections, with the exception of δ-sarcoglycan, was reported in the study by Sheriffs et al. [18], which also documented similar findings to our own regarding the expression of spectrin, dystrophin (rod domain and C-terminus), SGs (α, β, and γ), and β-dystroglycan (Table 5).

**Table 5:** Comparison of immunohistochemistry staining of FFPE sections between the present study and Sheriffs et al.

Antibody	Staining result of FFPE section	
	Present study	Sheriffs et al., 2001 [18]
Spectrin	Successful	Successful
Dystrophin (rod domain)	Successful	Successful
Dystrophin (C-terminus)	Successful	Successful
Dystrophin (N-terminus)	Unsuccessful	Successful
α-sarcoglycan	Successful	Successful
β-sarcoglycan	Successful	Successful
γ-sarcoglycan	Successful	Successful
δ-sarcoglycan	Not performed	Unsuccessful
β-dystroglycan	Successful	Successful
Dysferlin	Successful	Not performed
Merosin M-chain	Not performed	Successful
Myosin heavy chain (fast)	Not performed	Successful
Myosin heavy chain (slow)	Not performed	Successful

Our negative IHC staining for the N-terminus of dystrophin in FFPE muscle tissue is consistent with the findings of Hoshino et al. [17] but differs from the work of Sheriffs et al. [18], while our IHC analysis of the dystrophin rod domain and C-terminus is consistent with previous studies (Table 6). In practice, an antibody to the N-terminus of dystrophin faintly stained the sarcolemma of a fresh muscle specimen [25], and it is recognized that the epitope of some antibodies is destroyed or masked by fixation. The unsuccessful staining of the N-terminus of dystrophin in FFPE muscle in the present study therefore likely reflects epitope masking by the 10% neutral buffered formalin. The optimisation of an alternative fixative or antigen retrieval protocol could be considered for future studies.

**Table 6:** Immunohistochemistry staining of each domain of dystrophin using FFPE muscle sections in previous studies

Studies	Dystrophin IHC staining using FFPE muscle sections		
	Rod domain (DYS1)	C-terminus (DYS2)	N-terminus (DYS3)
Present study	Successful	Successful	Unsuccessful
Hoshino et al. 2000 [17]	Successful	Successful	Unsuccessful
Sheriffs et al. 2001 [18]	Successful	Successful	Successful
Sajid et al. 2010 [19]	Not performed	Successful	Not performed

*IHC i*mmunohistochemistry

A comparison of the methodology used in the present and previous studies [17–19] is given in Table 7. Citrate/EDTA buffer was the preferred pretreatment solution for the present technique, and was also used in the studies of Sheriffs et al. and Sajid et al. [18, 19]. The use of target retrieval solution as an antigen retrieval agent has also been documented [17]. The period of primary antibody incubation varies among studies, ranging from 15 min to overnight. In our study, 2 h of incubation with the primary antibody yielded no IHC staining of FFPE sections, either in the control or patient muscle samples on each slide (data not shown). Overnight incubation with the primary antibody was the optimal method that resulted in IHC staining equivalent to the frozen section. Moreover, FFPE sections of 3–5 μm are recommended to limit overlapping sarcolemmal staining.

**Table 7:** Comparison of methodology used for FFPE muscle in the present and other studies

Studies	Pretreatment buffer (duration)	Primary antibody incubation time	Thickness of FFPE sections (μm)
Present study	Citrate/EDTA (40 min)	Overnight	3 and 5
Hoshino et al. 2000 [17]	Target retrieval solution (20 min)	15 min	6
Sheriffs et al. 2001 [18]	Citrate/EDTA (4 min)	Overnight	8
Sajid et al. 2010 [19]	Citrate/EDTA (not stated)	2 h	8

## Conclusions

The successful IHC of sarcolemmal membrane-associated proteins in this study yields a promising tool for the diagnostic evaluation of common MDs when combined with clinical findings and changes in muscle biopsy, especially in situations where the fresh frozen technique is not routinely available. However, clinicians should be aware of the possible diagnostic pitfalls in patients with only a deficiency of the dystrophin N-terminus, indicating BMD. In cases with a strong clinical suspicion of a specific MD group, this technique will be beneficial in narrowing down the differential diagnoses leading to a subsequent genetic analysis. This IHC technique can be easily adapted by any pathology laboratory, even those with limited medical equipment, and its utility may be extended with the use of additional IHC markers. We encourage further studies to strengthen and standardize the use of this IHC technique for the analysis of FFPE muscle tissue.

## Abbreviations

BMD: Becker muscular dystrophy
DMD: Duchenne muscular dystrophy
FFPE: Formalin-fixed and paraffin-embedded
IHC: Immunohistochemistry
LGMD: Limb-girdle muscular dystrophy
LGMD1: Autosomal dominant LGMD
LGMD2: Autosomal recessive LGMD
MDs: Muscular dystrophies
SG: Sarcoglycan
